# “Not Just Anybody Can Do It”: A Qualitative Study of the Lived Experience of Inpatient Palliative Care Professionals in China's Mainland

**DOI:** 10.1089/pmr.2021.0014

**Published:** 2021-04-27

**Authors:** Rui Fu, Jia Lu Lilian Lin, Jianjun Jiang, Tingting Zhou, Jay Pan, Peter C. Coyte

**Affiliations:** ^1^ Institute of Health Policy, Management and Evaluation, Dalla Lana School of Public Health, University of Toronto, Toronto, Ontario, Canada.; ^2^ HEOA Group, West China School of Public Health and West China Fourth Hospital, Sichuan University, Chengdu, China.; ^3^ Institute for Healthy Cities and West China Research Center for Rural Health Development, Sichuan University, Chengdu, China.

**Keywords:** health personnel, inpatients, palliative care, palliative medicine, qualitative research

## Abstract

***Background:*** Over the past 5 years, China has invested substantially in palliative care programs to meet the rising demand for such services. In China's mainland, most palliative care programs are embedded within an established hospital unit, but a small subset of providers practice exclusively in a stand-alone inpatient palliative care department.

***Objective:*** To explore the lived experience of professionals at an independently operating palliative care hospital department in China's mainland.

***Design:*** We used purposive sampling to select palliative care physicians and nurses. Semistructured in-depth interviews were conducted in person. Thematic analysis was used to elicit key themes that pertained to participants' lived experience.

***Setting/Subjects:*** Ten palliative care physicians and seven nurses at the palliative medicine department in the West China Fourth Hospital of Sichuan University in Chengdu, Sichuan Province, participated in the interviews.

***Results:*** Three themes related to participants' lived experience were (1) interactions with patients and families (e.g., frequent encounters with death, communication difficulties, witnessing family struggles, and developing mutually trusting relationships); (2) factors influencing their work life (e.g., supportive working environment, unmet training needs, policy restrictions, and lack of public awareness); and (3) perceived nature of work (e.g., complex and demanding, underappreciation, encroachment of work stress into personal life, deriving accomplishment from work, and personal growth).

***Conclusion:*** This study helps fill the void in the palliative care literature regarding the lived experience of inpatient palliative care professionals in China's mainland. Our findings revealed factors influencing the well-being of palliative care professionals that are meaningful to policymakers.

## Introduction

Over the past 5 years, China has invested substantially in palliative care to meet rising demand.^1^ After the introduction of the first national palliative care guideline in 2017,^2^ a nationwide pilot program was launched and expanded to 71 regions by the end of 2019.^3^ Despite these recent advancements, there are only 12 palliative care units operating in China's mainland to serve a population of 1.4 billion, compared with 46 such units in Japan and 29 in South Korea.^4^ Furthermore, although efforts have been made to expand health insurance coverage in China, the scope of services covered and the degree of financial protection still lag far behind those in many low- and middle-income countries.^5^ This is especially applicable to palliative care considering its nascent status.^6^

A lack of palliative care professionals has been identified as a major barrier to its development in China's mainland.^7^ Meanwhile, research on the experiences of palliative care professionals is also scarce.^8^ The six existing studies on palliative care providers in China's mainland documented oncology nurses and physicians who provided such care,^9–11^ or assessed the perceptions of palliative care among other health care professionals.^12–14^ A void in the literature concerns the experiences of professionals who practice exclusively in a stand-alone inpatient palliative care ward. Most palliative care units in China's mainland are embedded within established oncology departments rather than operating independently.^7^ As hospitals remain the main setting for death for Chinese older adults,^15^ increased demand for this care setting may warrant expansion of autonomous inpatient palliative care units. Thus, it is important to gain an understanding of the operation of independent palliative care units through the perspectives of professionals who practice at these locations.

Studies from Hong Kong and Taiwan have shed light on the lived experience of physicians and nurses who work in a hospital unit dedicated to palliative care. A spectrum of emotional burdens such as helplessness,^16–18^ guilt,^19^ and suffering^20^ can arise from frequent encounters with death. Difficulties with separating work life from personal life are common, as some palliative care professionals report personal traumas (such as the loss of a parent) being evoked during work^20,21^ undergoing radical overhauls of core belief systems as a result of their work.^19,21^ As health care professionals, these individuals report low self-competence,^22^ unmet work expectations,^17^ poor work continuity,^23^ and an ambiguous sense of professional identity.^21^ Although it is likely that palliative care professionals in China's mainland share certain overlapping experiences with their counterparts in Hong Kong and Taiwan, no study has been conducted to verify this hypothesis. Furthermore, due to the institutional environment in China's mainland and the limited public awareness regarding palliative care,^13^ local professionals may face different obstacles that have yet to be documented.

We address these literature gaps in this study. To the best of our knowledge, this is the first qualitative investigation of the lived experience of professionals in a stand-alone inpatient palliative care department in China's mainland. We sought to capture the salient experiences of these professionals to identify the unique barriers and facilitators to their well-being.

## Methods

### Design and setting

This qualitative study was conducted at the palliative medicine department (henceforth “the Department”) at the West China Fourth Hospital of Sichuan University, a large teaching hospital located in Chengdu, Sichuan Province. Founded in 1996, the Department has the oldest independently operating inpatient palliative care unit in China's mainland,^7^ which functions as the only palliative care program in Sichuan Province with a population of 84 million.^24^ For comparison, Sichuan Province is just over four times larger than the state of Pennsylvania in land area, has almost double the population density, and is 40% rural compared with 27% for Pennsylvania.^25^ In July 2019, full-time employees who delivered palliative care exclusively at the Department were 15 physicians and 28 nurses, as well as social workers and drivers. Its inpatient ward has 60 beds that accommodate 1500 patients (aged 0–107 years) annually.^26^ This study received ethical approval by the West China School of Public Health, Sichuan University on July 26, 2019 (Supplementary Appendix S1), and was conducted in accordance with the ethical principles comprised in the Declaration of Helsinki.^27^

### Sample

In July 2019, we used purposive sampling to identify physicians and nurses who were in regular contact with patients and patients' family members. This yielded 32 candidates (12 physicians and 20 nurses) and all were invited by email to participate. Seventeen providers (10 physicians and 7 nurses) accepted the invitation and those who declined reported time conflicts (*n* = 8), lack of interest (*n* = 2), or discomfort with being interviewed (*n* = 3); 2 people could not be reached by email. The lead researcher (R.F.) obtained written informed consent (Supplementary Appendix S2) from participants after thoroughly explaining the study, including the availability of a social worker referral after the interview.

### Procedure

In August 2019, the lead researcher (R.F., female PhD candidate with training in qualitative interviewing), accompanied by a research assistant (T.Z.), conducted semistructured interviews in Chinese with each participant in the conference room of the Department. T.Z. took notes of nonverbal cues during the interviews. An interview guide was used (Supplementary Appendix S3) and updated throughout the data collection process to embed newly emerged themes. Gentle probing explored participants' felt experiences and personal meanings in greater depth. Each interview lasted 30–60 min and was audio recorded. R.F. anonymized and transcribed audio recordings verbatim. Transcripts were translated into English and verified for accuracy by J.L.L.L.

### Data analysis

Guided by Braun and Clarke's six phases of thematic analysis,^28^ a rigorous yet theoretically flexible approach to analyzing qualitative data, R.F. and J.L.L.L. coded, identified, and analyzed themes through a recursive and reflexive process. Deidentified transcripts were imported into QSR NVivo 12 for management and analysis. After familiarization, R.F. and J.L.L.L. inductively coded all transcripts line by line and developed an initial coding structure. Through comparing patterns across interviews, searching for contradictions, and grouping and regrouping concepts, the coding structure was continually refined. All authors developed and refined themes and subthemes through discussions and contextualizing the analysis in relation to the practice setting and existing literature.

## Results

### Participant characteristics

Among the 17 participants, 10 (58.8%) were physicians and 7 (41.2%) were nurses (Table 1). Their median age was 34 years (range, 24–52 years) and most were women (88.2%), married (76.5%), and had children (70.6%). Most participants reported the absence of formal training in palliative care (82.4%) and had worked in a nonpalliative care hospital unit before joining the palliative medicine department (76.5%). The duration of employment in this Department ranged from 1 month to 14 years with a median of 5 years.

**Box 1.** Interactions with Patients and Families

Subthemes	Quotation(s)
1.1. Frequent encounters with dying patients	Because I'm quite emotional, it's been very difficult, especially at the beginning, to work with these patients who are so severely ill and visibly suffering. My initial experience was extremely uncomfortable, almost too painful for me to bear. I felt really bad to be in close contact with these terminally ill patients every day. It took me about two weeks to adapt [to this work environment], and I had to slowly adjust myself over time. (N6) Seeing death all the time, it's so difficult. Even though so many patients die in our department, and they aren't members of our own family, but after a long period of time, the stress adds up, and there is actually no way for us to relieve the stress. We just have to deal with it on our own. It feels terrible. (N3) A lot of us couldn't bear seeing so many dying patients for a long period of time. It was most difficult with pediatric patients. (D2) The most stressful part of the job is caring for young patients—I think young adults are okay—but it's particularly challenging with children! When I was unmarried and didn't have kids, I didn't feel this way. Back then if a child patient came and I was supposed to give them a shot, I would do it, although I'd get very sad when I see them cry. But ever since I had my own baby, I cannot bear to see any child patients! Every time I see a child here, I cry! I do everything possible to avoid giving these children shots. But eventually I have to, because the work falls on me. (N1)
1.2. Having to withhold diagnoses from patients	We definitely will not tell the truth to the patient, but we must tell the truth to the patient's family members…In China, we still don't tell anything to the patient. We communicate mostly with their family members. If somehow the patient found out about their condition and couldn't handle it, their family members would make so much trouble for us. (D3) We've had patients who had no idea about their own health condition, so you had to discuss everything with their family members…It made me really sad because he had such a terrible condition, but you couldn't let him know, and he didn't know either. Had he known he might have wanted to discuss end-of-life arrangements, but that opportunity was taken away from him. (D4)
1.3. Witnessing the emotional and financial struggles of patients' family members	The most stress for me comes from the emotional reactions of patients and family members. Sometimes they are in denial that their loved one is dying, and many of those situations are difficult even for us to take in, let alone family members. (N7) Many patients in the end are incurable, but their families keep spending money on treatment. Especially for cancer patients, at the end of chemotherapy, the family spent up almost their entire savings, yet they couldn't save the patient. I see this so often in my practice. See, you've spent so much money, but in the end they still died. I understand how these families feel, but unfortunately, we can't do anything about it. (D5) Although his suffering ended, there were many issues that his family had to handle after he died. It was very depressing, and I felt sad with the family. (D2)
1.4. Accommodating families' financially driven care decisions	One of our pressures comes from family members' lack of understanding. This lack of understanding is mostly due to their financial situation, since the choices available to them are bound by financial constraints. Many of the palliative care services are not covered in the public insurance scheme, so families have to pay out-of-pocket for most of the services we provide. That obviously puts a lot of pressure on families, and their goal is to restore their family member's health while spending the least amount of money. Because of this, they are often skeptical and antagonistic towards doctors, which puts a lot of pressure on us. It would be ideal for me as a doctor to only focus on the medical piece and not the financial piece. But for us, we have to take into account multiple factors, including the patient's condition, the cost of treatment, and the family's financial ability. (D2)
1.5. Dealing with uncooperative family members	Many family members do not understand our job…Well, they've already spent a long time seeking treatment from many different facilities before coming here. Once they arrive [at our department], the family members themselves are like half-doctors, acting like they know everything. They don't respect our nursing staff and often question what we do. This is the kind of environment where we do our job…Most of the time we have to concede to family members so that conflicts can be resolved. (N3) The patient had bone metastasis…she was suffering from a lot of pain…the family did not want to pay out-of-pocket so I changed her pain medication to an oral medication and had to increase the dosage to offer sufficient pain relief. The patient's sister was strongly opposed to the high dose. […] When I did the rounds the next morning, I saw the patient in so much pain that she couldn't move… I tried to convince her sister that we have to believe the patient when she says she's in pain. The sister said no! Her pain can't be that bad! We often encounter such family members who are so uncompromising in their beliefs that we cannot convince them even after repeated attempts to communicate. That makes it impossible to implement our treatment plan because we have to worry about family members causing a big kerfuffle. (D5)
1.6. Developing mutually trusting relationships	The patient was initially in pain, and he held a distrustful attitude towards us. As soon as he came in, he revealed a sense of distrust in his every word. But after we offered him a thorough explanation, and once his condition improved the next day, his attitude took a complete turn. Our mood improved as well…because we felt that our efforts were appreciated so that made us feel better. (D2) I took over the patient when she was transferred to our department. Her mother began to become more accepting of her daughter's condition, and started to feel that death would be a relief …I communicated with the family daily. After the girl's death, her mother talked with me for a long time. She expressed her gratitude for the services of our department and the attentive attitude of our medical staff. She felt grateful to us for providing high quality care to her daughter in her final days. (D7)

**Box 2.** Factors Influencing Work Life

Subthemes	Quotation(s)
2.1. Positive work environment	Our department has a good environment, and everyone helps each other. The leaders are also very kind and caring, so newcomers to our department should find it an enjoyable place to work. (D4) I think the leadership has been doing a good job at helping us relieve stress. For example, the director of our nursing department has a very good relationship with us. (N5)
2.2. Unmet needs for skill training	For the patients who come to our Department, some family members have told us that they weren't only hoping to receive palliative care services, but that they were also looking for some kind of spiritual support or mental health counselling. I feel that our Department is still lacking in this area, because we don't have any psychiatrists on staff who's specially trained to be able to actively intervene. (D1) In Taiwan, they do a very good job of providing human services, especially mental health counselling for patients. We have not undergone professional training, but we are often learning their concepts and methods. I personally think that, because I'm not that familiar with patient relations, but I know that psychiatrists [in China] who work in this field are not doing a good job either! They can't do it well, because their knowledge doesn't seem to be useful in our Department. As for us, we have a lot of clinical experience, but when it comes to medical knowledge in the field of psychology, we are deficient. (D9)
2.3. Lack of special policies supporting palliative care	The medical insurance performance requirements, such as the obligation to provide a certain level of public insurance-covered health interventions, are perhaps the biggest source of annoyance for us. We not only have to face patients, but also have to adhere to the hospital's medical insurance requirements. We must implement these requirements and report on our performance on a regular basis. For example, we must explain why the antibiotic prescription rate in our department is so high, and why our department's mortality rate is so high compared to other departments. (D8) Many of the services we provide, such as bathing for critically ill patients and aromatherapy, have not been approved by the Price Bureau to include in the hospital fee-for-service list. We've been very keen to expand our aromatherapy services, because these treatments are very effective for our patients, but after submitting applications to the Price Bureau year after year, we could not expand these treatments because our applications to the Price Bureau to include aromatherapy in the hospital fee-for-service list have been denied every time. (D5)
2.4. Limited public understanding of palliative care	Many people see our department as a place where patients await their death. They question the need for this place, they think that nothing happens here while patients wait to die. If the patient's condition is fatal and nothing more can be done to save their life, why spend all that money here? A lot of people can't understand because they think these patients are going to die regardless. (D3) Our department used to be called hospice and end-of-life care. Many patients got nervous and afraid when they heard this name, because they knew it meant that death was near. Subsequently, our department was renamed to palliative care. When patients heard the word “care,” they thought our services were no different than nursing care. Now our department name has been changed to palliative medicine. Patients seem less resistant to the term “medicine” so it is better. (N4)

**Box 3.** Perceived Nature of Work

Subthemes	Quotation(s)
3.1. Nature of work as complex and demanding	Many nurses in our department wanted to transfer to a different department, they didn't want to stay here. Maybe everyone felt under pressure, in all aspects…because our specialty is indeed very complex! Patients will call you over each time they have some pain here or itch there, so you are always running around. I just feel that, after working here for over ten years, it's such a messy and stressful environment. (N1) Now that I have worked here for many years, having had close contact with so many patients and families, I understand how this experience is nothing like what I had expected prior to coming here. I realized that the work isn't so simple at all…even if you receive years of training in palliative medicine, it's not a given that you will become a competent palliative care professional. (N6) In addition to the foundational medical ethics, you should have a strong sense of responsibility to patients, and a positive attitude. You have to consider that the patient is terminally ill, and their family members are suffering immensely. Your attitude towards them must be very kind, gentle, patient, responsible, and you have to explain the disease to them slowly and thoroughly and be ready to take responsibility for dealing with any issues that arise. (D4)
3.2. Limited understanding of the profession from outsiders	People are very curious when I tell them about my job. Everybody knows about specialties like internal medicine, surgery, and so on, so when I mention palliative medicine, their first reaction is “what is that?” Then I say palliative care, but they still have no clue. When I finally mention hospice care, they usually respond with “oh so you are dealing with the dead” (N6). Last week the director of an oncology department visited our ward and said our treatment plans are good, but our work is too unfulfilling! Well, I said to him, maybe everyone has a different understanding of how to derive a sense of accomplishment (D5). It's arduous work that's highly stressful but doesn't pay well. (D2)
3.3. Deriving accomplishment from work	We once had a patient who had such severe pain at home that he wanted to jump off from the apartment building. Then he had come to us and we helped relieve his pain. After that, he could eat and sleep again, and he said his quality of life had improved to a level similar to a normal person, which was an enormous improvement. So being able to offer great satisfaction for patients made my job worthwhile. (D4) I think the greatest sense of accomplishment for me is allowing patients to pass away with little pain or discomfort…I think that as long as I can help to lessen the pain for my patients and their families, even if just by one percent, it's still an achievement, right? (D5) I feel quite accomplished when I think about my job, because the truth is, this job is not something that anybody can do. (N6)
3.4. Encroachment of work stress into personal life	We face all kinds of pressures—psychological stress, financial burden, pressures coming from everywhere. I think the most important part is the psychological stress. (N6) The work stress gradually accumulates over time. If you stay in this position for too long, you will feel irritable. There is a lot of pressure coming from all aspects, from the lack of understanding from patients, the lack of understanding from their family members, and not being able to balance my own family life due to work stress. (N2)
3.5. Personal growth by viewing death as less taboo	The way we feel about patients in our department is different from how other departments see their patients. Sometimes we feel that death would be a true relief for the family members, because I don't think there's a need to force anything with these patients. (D8) I don't espouse “continuing the chemotherapy as long as you're alive.” (D5)

**Table 1.** Characteristics of Participating Palliative Care Professionals

Characteristics	Counts (total* n* = 17)	%
Gender		
Female	15	88.2
Male	2	11.8
Age (years)		
Mean (SD), years	33.4 (6.4)	
Median (IQR), years	34 (7)	
Range, years	24–52	
Marital status		
Single	4	23.5
Married	13	76.5
Children		
Has child(ren)	12	70.6
Without children	5	29.4
Native of Chengdu, Sichuan		
Yes	7	41.2
No	10	58.8
Training		
Physician	10	58.8
Nurse	7	41.2
Received formal training in palliative care		
Yes	3	17.6
No	14	82.4
Years at the department		
Mean (SD), years	5.7 (5.2)	
Median (IQR), years	5 (1)	
Range, years	0.08–14	
Professional experience before joining the department		
Intensive care unit	1	5.9
Oncology	2	11.8
Endocrinology	2	11.8
Geriatrics	3	17.6
Obstetrics and gynecology	1	5.9
Internal medicine	1	5.9
Palliative care	1	5.9
General nurse	1	5.9
Administrative nurse	1	5.9
No prior professional experience	4	23.5

IQR, interquartile range; SD, standard deviation.

### Thematic analysis results

We identified three main themes representing the lived experience of participants. We summarize these themes hereunder with quotes presented in Boxes 1–3.

#### Interactions with patients and families

Participants spoke at length about their interactions with patients and family members (Box 1). Immense psychological distress was reported as a direct result of frequent encounters with dying patients. Participants described caring for pediatric patients as the most challenging part of the job; particularly, female nurses reported such experience to be emotionally draining and unbearable after they became mothers themselves.

Participants reported hiding the diagnosis from the patient at the request of the patient's family even when doing so generated intractable moral distress in themselves. Moreover, participants faced criticism, doubt, and even confrontation from families facing financial stress regarding patients' treatment plans. Participants shared that when dealing with distrustful, opinionated, or uncooperative family members, a common strategy employed was to concede to family members so that conflicts could be resolved.

Participants discussed empathizing with patients' families through witnessing their grief and financial struggles. They reported feelings of despair and powerlessness in knowing that families had exhausted their savings and that even after the death of the patient, family members were suffering from financial and emotional burdens.

Many participants reported positive experiences where they saw a shift in the orientation of patients and families from being initially anxious and stubborn to appreciative and calm. Some participants reported maintaining contact with their past patients' family members for a long time after the patient's passing.

#### Factors influencing work life

Factors at various levels influenced participants' work life (Box 2). Within the Department, participants reported having supportive leaders and colleagues to help them alleviate work stress. They described a lack of training opportunities to develop skills to support the mental health of patients and family members.

At the policy level, participants voiced concerns regarding the mismatch between the idiosyncrasies of palliative care and the one-size-fits-all policies that govern the insurance caps on most palliative care services. Participants reported advocating for specialized services (e.g., aromatherapy) to be listed under reimbursable services to enhance patient choice and satisfaction.

At the sociocultural level, participants reported limited public awareness and acceptance of palliative care as some patients' family members questioned the rationale for paying largely out of pocket for such care. Interestingly, some participants described a positive shift in patients' and families' attitudes when the name of the Department was changed from “Hospice and End-of-Life Care” to “Palliative Care” and eventually to “Palliative Medicine” as the term “medicine” was commonly associated with professionalism and expertise.

#### Perceived nature of work

Participants detailed how they perceived their work and how their work had impacted their personal lives (Box 3). They described having an overwhelming workload with a level of complexity that significantly exceeded their expectations. Physicians discussed how their work demanded highly sought-after qualities including tenacity, compassion, and perseverance. Participants reported feeling underappreciated by peer physicians practicing in other disciplines. When speculating about the reason for poor recruitment and retention of palliative care professionals, participants felt that aside from suffering from immense physical and psychological work stress, they were also undercompensated.

Participants reported three main mechanisms through which they gained a sense of work accomplishment. First, being able to reduce bodily pain for patients and to optimize their quality of life. Second, enabling patients to die with dignity. Lastly, by just being a palliative care professional *per se* as it was work that is not something that anybody can do.

Participants reported stress accumulating from the encroachment of work into their personal lives, inducing a sense of powerlessness and increased irritability. They also described experiencing a shift in their own belief systems as they began to view death as a natural experience with less taboo. They explained that unlike their peers from the oncology and critical care departments, they held a more accepting view of death and would wish for their family and friends to receive palliative care rather than aggressive life-sustaining treatment during the last phase of life.

## Discussion

### Main findings

This study is the first analysis on the lived experience of palliative care professionals based at a stand-alone palliative care hospital department in China's mainland. The psychological stress reported by our participants due to frequent encounters with death is consistent with observations from Hong Kong and Taiwan.^16–22^ We found female nurses with young children faced the greatest emotional challenge when caring for a dying young patient, whereas female nurses with older children and physicians of both genders reported less distress. This finding expands the literature concerning ethnically Chinese palliative care providers in two ways: first, in line with prior studies already suggesting that caring for pediatric patients causes exceptionally high stress for nurses,^19,21^ we provide an explicit description of those nurses who are particularly susceptible to such stress; second, although previous studies have shown that nurses often confronted traumatic personal memories at work,^16,20–22^ our findings suggest that even positive personal experiences (such as having a baby) can trigger negative emotions if evoked at work. Since no male nurses were included in this study, future research should assess whether the same impact of providing pediatric palliative care applies to male nurses.

Participants reported withholding diagnoses from patients at the request of their family members. This practice is in line with a cultural norm, wherein a terminal diagnosis is conveyed to the oldest or head of the family first and sometimes not disclosed at all to the patient.^13^,^29^ A novel finding of our study is that while withholding diagnoses from patients complicates participants' care provision, it is this missed opportunity for advanced care planning that leads them to feel guilt and long-lasting regret. This is in contrast to the experiences of palliative care professionals in Hong Kong who reported fostering hope in patients by facilitating conversations between patients and their families to find solutions to unfulfilled family responsibilities.^18^ China's mainland has yet to enact legislation to formalize palliative care procedures, especially in ensuring advanced care planning. In comparison, two pieces of legislation—the Natural Death Act (2000) and the Patient Autonomy Act (2016)—have been launched in Taiwan to safeguard the rights of patients. Palliative care professionals reacted positively to these pieces of legislation as they helped to streamline care duties and clarify care goals.^30^ In the absence of mandatory regulations and quality standards, palliative care professionals often face ethical dilemmas due to uncertainties in their practice, which adversely impact their well-being.

In our study, families that faced financial pressures often raised issues with participants by questioning them or refuting their recommended treatment plans. The difficulties posed by financially driven care decisions have not been explicitly examined in Hong Kong- and Taiwan-based studies due to the presence of different insurance schemes and economic structures.^31^,^32^ Hence, this could be a unique stressor faced by palliative care professionals in China's mainland. Owing to the uniqueness of palliative care, some services tend not to be included in the benefit package of the Chinese social basic health insurance programs, meaning that some patient copayments would always be required.^33^ In our context, this means that conflicts arising from an absence of financial protection may be inevitable between family members and palliative care professionals. Therefore, although efforts must be made to expand the list of palliative care services in insurance programs and increase the reimbursement rates, interventions such as peer support groups are needed to help palliative care professionals resolve conflicts with families and regain their ability to provide optimal care.^17^

Prior studies have often found palliative care professionals, especially nurses, to have poor self-competence and to lack a sense of accomplishment.^16,21,22^ These studies also revealed common ways in which palliative care professionals gained work accomplishment, such as through improving patients' quality of life and ensuring their dignified death. A unique finding of our study is that “not just anyone can do it” constitutes a distinct source of accomplishment for some participants. This is consistent with a Taiwanese study that found that some palliative care nurses regard their work as a privilege as it allows them to encounter their inner selves, establish authenticity, and prepare for their own death in a positive manner.^20^ We expand on those findings by suggesting that the distinct sense of meaning and worth that palliative care professionals derive from their work may be a result of fully understanding what the work entails. These findings imply that reinforcing the distinctive role of palliative care and strengthening the professional identity of these professionals may be an effective way to help them maintain their commitment to work. As such, the scarcity of skills training opportunities identified by the participants—such as a lack of curriculum for providing mental health counseling to patients and families—should be addressed to expand their professional skillset, thereby bolstering their professional identity.

### Strengths and limitations

The Department we focused on is the oldest and one of the largest inpatient palliative care units in China's mainland,^7^ and our participants are among the first group of professionals to practice at a hospital department dedicated to palliative care. This unique setting allowed us to draw novel conclusions about this understudied population.

Our study has limitations. First, the study's relatively small sample may limit the transferability of the findings to other settings. However, our sample represents over half (53%) of all palliative care professionals at the Department, including 83% of physicians. Hence, we believe that by conducting in-depth interviews, our findings likely reflect the dominant experiences of these inpatient palliative care professionals. Second, the lived experience of nurses may have been underrepresented in our analysis since physicians outnumber nurses in our sample. However, we observed that experiences among nurses were similar while physicians' experiences were more heterogeneous. Third, our sample is skewed toward women with no male nurses, which is consistent with the gender imbalance of the nursing workforce in China.^34^ Fourth, due to the large Chinese population and a tiered health care delivery system with wide variations in hospital scale and function,^35^ findings of this single-center study may not generalize to other parts of China or internationally. Lastly, our interviews were conducted before the coronavirus disease 2019 pandemic that has had major impacts on the lives of frontline health care workers, including those delivering palliative care. As such, we will reinterview participants to understand how their experiences have been impacted by this pandemic.

### Recommendations

Continuing education opportunities for palliative care professionals to improve communication skills, mental health counseling ability, and advanced care planning needs to be expanded. These interventions should take on diverse forms including courses, real-world application (e.g., role-playing exercises^22^), and reflective activities.^16–18,20–22^ Intradepartmental programs such as stress and emotion management workshops^36^ need to be provided. Those who are tasked with caring for pediatric patients or who have young children warrant additional resources and institutional support. On a broader scale, a systematic approach that includes promoting public awareness of palliative care and developing palliative care-specific training curriculum is needed to address the root causes of the scarcity of the palliative care workforce and their work-related burden. Specifically, regulatory bodies need to include palliative care services with demonstrated effectiveness in the list of reimbursable services and expand reimbursement rates of these services.

## Conclusion

China is an important case study of inpatient palliative care programs. In this study, we interviewed a group of physicians and nurses at an independent palliative care unit in a large teaching hospital in Sichuan Province to understand their lived experience and self-identified barriers and facilitators to their emotional and physical well-being. Findings of this study have actionable implications for human resource policies and other interventions to help balance the daily living of these professionals while ensuring their quality of care.

## Supplementary Material

Supplemental data

## Abbreviations Used

IQR: interquartile range
SD: standard deviation
