# Supplementary material for: “Not Just Anybody Can Do It”: A Qualitative Study of the Lived Experience of Inpatient Palliative Care Professionals in China's Mainland
Source: Palliat Med Rep. 2021 Apr 27;2(1):104–12. doi: 10.1089/pmr.2021.0014 (PMC8241397; doi:10.1089/pmr.2021.0014)
Supplement: Supplemental data [file Supp_AppS1.docx]

“Not just anybody can do it”: a qualitative study of the lived experience of inpatient palliative care professionals in China’s mainland

**Supplemental Information**

**Table of Content**

| **Appendix S1** Ethical approval letters in English and Chinese | **1** |
| --- | --- |
| **Appendix S2** Written informed consent letters in English and Chinese | **4** |
| **Appendix S3** Interview guide (translated into English) | **8** |

**Appendix S1** Ethical approval letters in English and Chinese


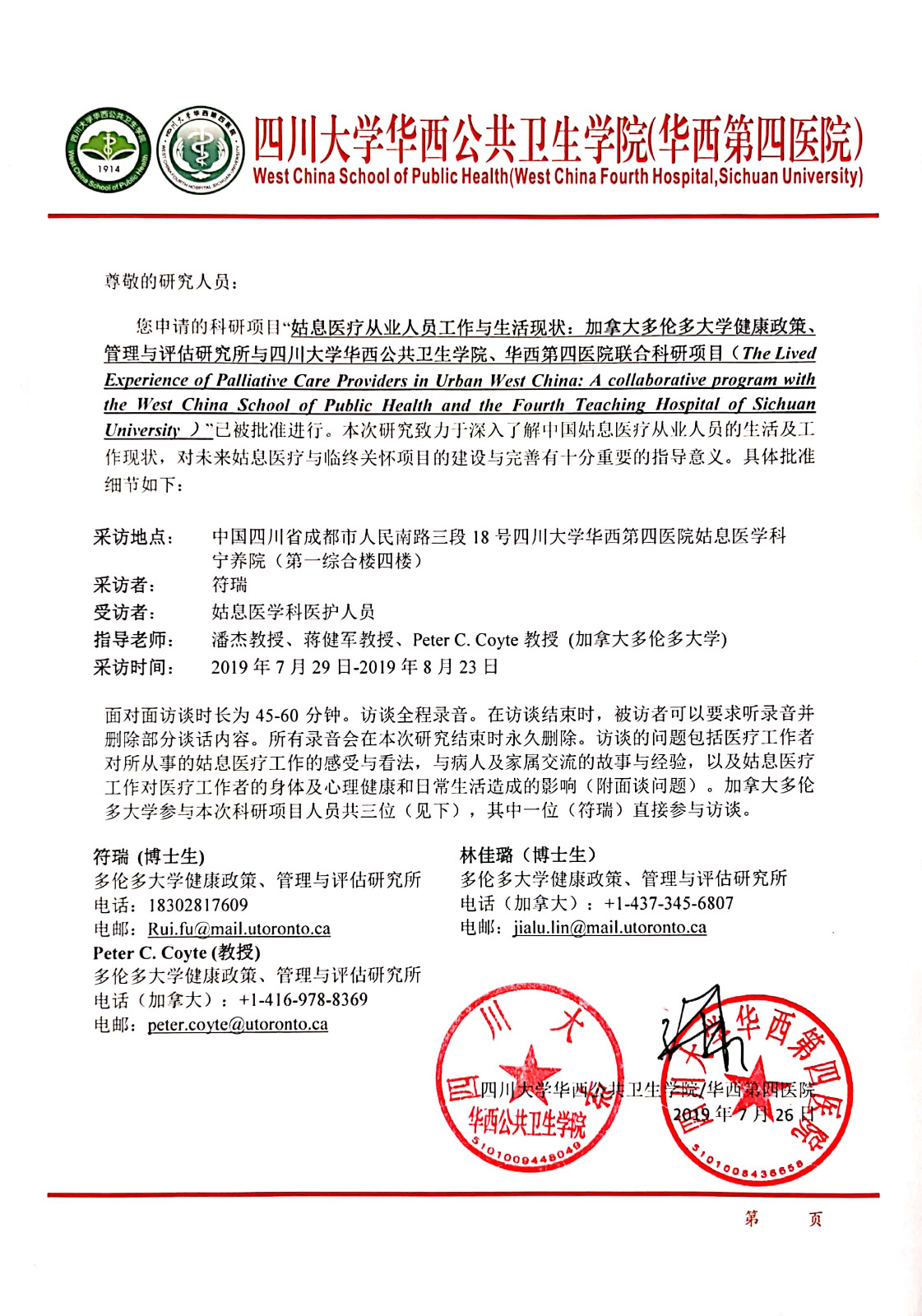


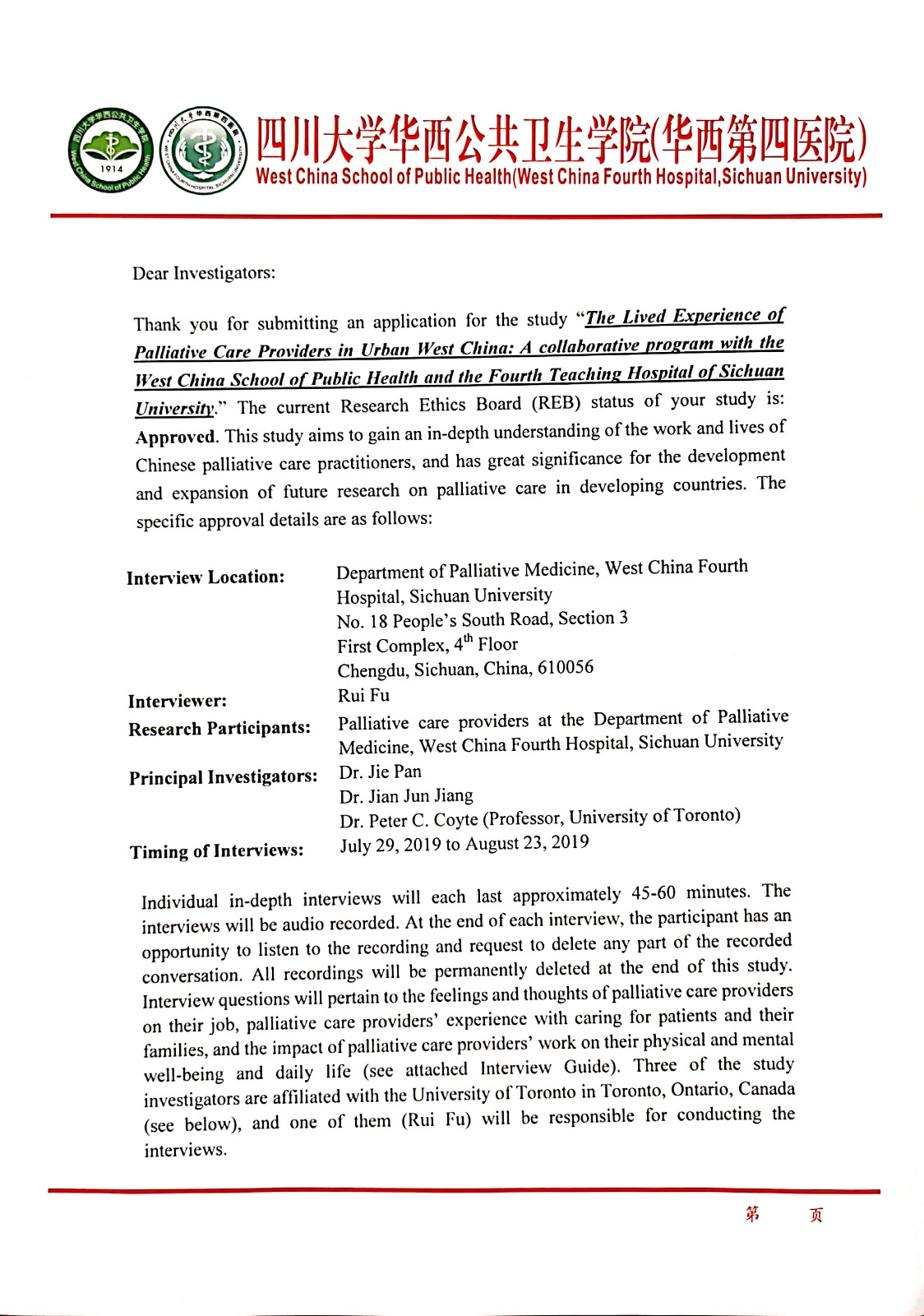


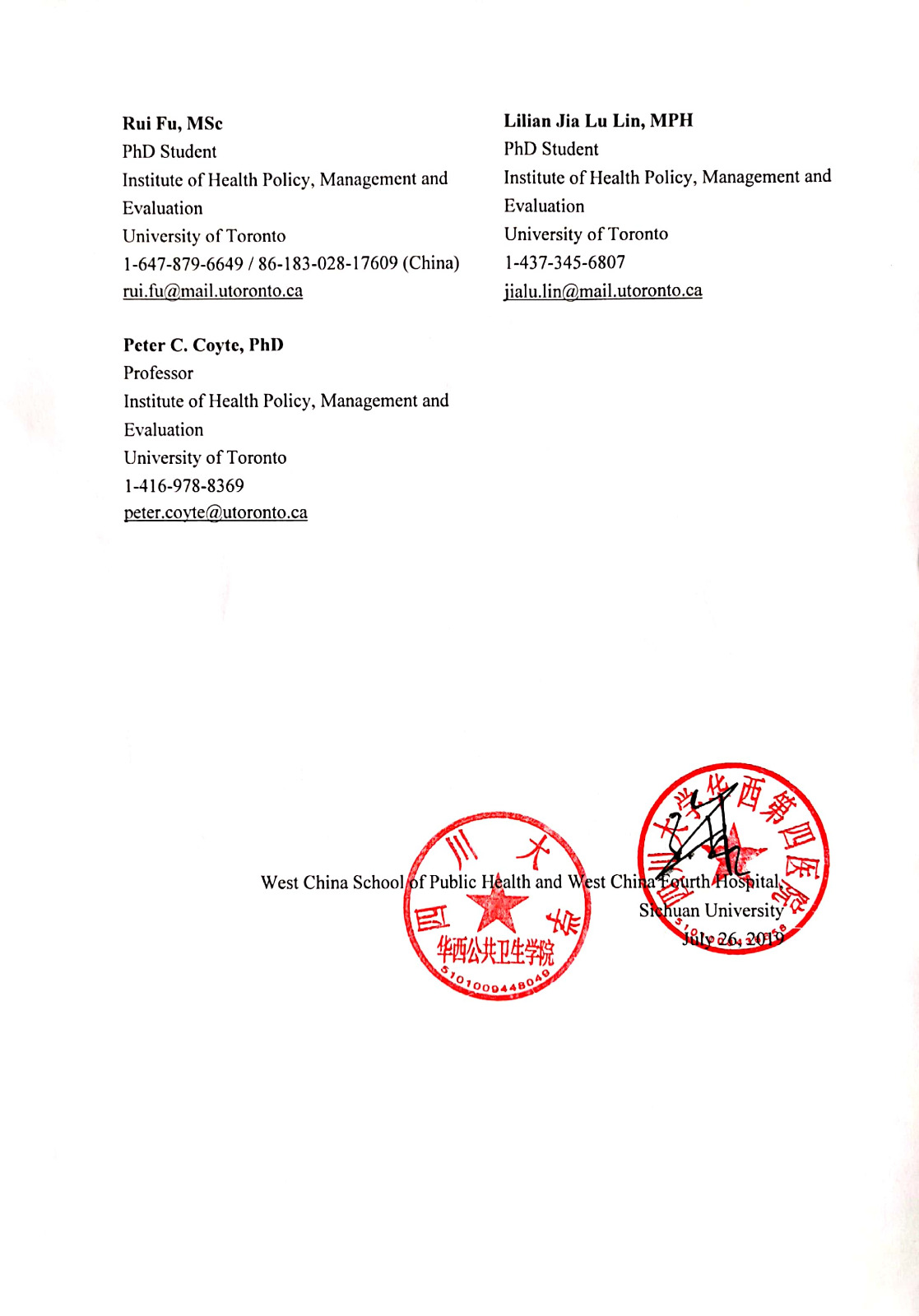


**Appendix S2** Written informed consent letters in English and Chinese

**参与研究的知情通知及授权书**

**研究题目：**

姑息医疗从业人员工作与生活现状：加拿大多伦多大学健康政策、管理与评估研究所与四川大学华西公共卫生学院、华西第四医院联合科研项目

**A. 研究人员**：

| 符瑞 (博士生)  多伦多大学健康政策、管理与评估研究所  电话：18302817609  电邮：[Rui.fu@mail.utoronto.ca](mailto:Rui.fu@mail.utoronto.ca)  潘杰（教授、副院长）  四川大学华西公共卫生学院  电话：85501272  电邮：[panjie.jay@scu.edu.cn](mailto:panjie.jay@scu.edu.cn)  Peter C. Coyte (教授)  多伦多大学健康政策、管理与评估研究所  电话（加拿大）：+1-416-978-8369  电邮：[peter.coyte@utoronto.ca](mailto:peter.coyte@utoronto.ca) | 林佳璐（博士生）  多伦多大学健康政策、管理与评估研究所  电话（加拿大）：+1-437-345-6807  电邮：[jialu.lin@mail.utoronto.ca](mailto:jialu.lin@mail.utoronto.ca)  蒋建军（教授、副主任）  四川大学华西第四医院姑息医学科  电话：85501520  电邮：[jiangjianjun@scu.edu.cn](mailto:jiangjianjun@scu.edu.cn) |
| --- | --- |

**B. 研究目的**

您现在被邀请自愿地参加一项研究。这份同意／授权书包括有关这项研究的资料。这项研究的目的是要了解中国姑息医疗及临终关怀医护人员的工作性质、生活现状，以及工作对职业认知、身体健康状况以及生活质量的影响。我们想要知道这些医疗工作者对于工作的看法，选择从事姑息关怀的原因，以及如何平衡工作与个人生活。您被邀请参加这项研究是因为您目前是四川大学华西第四医院姑息医疗科的医生或者护士。

**C. 访谈流程**

如果您同意参与这项研究，我们将对您进行访谈，流程如下：

- 访谈将面对面进行。
- 访谈地点位于华西第四医院内。
- 我们会将访谈的过程录音并做笔记。在访谈结束时，您可以要求听录音并删除部分谈话内容。所有录音会在本次研究结束时永久删除。
- 访谈的问题包括您对所从事的姑息医疗工作的感受与看法以及您与病人及家属交流的故事与经验。
- 访谈的问题还包括姑息医疗工作对您的身体及心理健康和日常生活造成的影响
- 访谈时间为45-60分钟。

**D. 隐私维护**

为保护您的隐私权，我们采集的所有信息都会被谨慎保护。所有与本次研究相关的纸质资料会被安置在四川大学华西公共卫生学院内一个上锁的办公室内。所有电子资料（包括访谈录音）会被保存在一个加密电脑中的加密文件夹里。所有的访谈会在一个私人房间（办公室）内进行。您提供的内容绝不会被用于辨认您的身份或者在使用中透露您的身份。

**E. 潜在的危险及不适**

您作为受访人的身份不会被上述研究人员之外的人其他人知晓。在访谈中您可以选择不回答让您不舒服、不方便的问题。虽然我们不认为本次访谈会有明显的潜在风险会给您带来不适，但您一旦觉得我们触及到了您不想回答的敏感话题，可以随时打断访谈并选择不回答。如果您在访谈后出现焦虑或者其他不适，我们非常乐意提供心理疏导服务。

**F. 潜在的利益**

本次研究的结果会帮助我们更深入地了解中国姑息医疗从业人员的生活及工作现状，对未来姑息医疗与临终关怀项目的建设与完善有十分重要的指导意义。

**G. 参与这项研究之外的其他选择**

参与本次研究是您的自愿选择。您可以在任何时间选择退出，撤回您的授权。如果您退出本次研究，我们会将您的所有参与资料永久删除。您不参与本次研究的决定不会对您在四川大学华西医院的工作带来任何形式的不良影响。

**H. 联络信息**

如果您对本次研究还有其他疑问或者想要这次研究的相关出版物（中英语），请联系符瑞（微信二维码见下一页；电话：18302817609; 邮箱：[rui.fu@mail.utoronto.ca](mailto:rui.fu@mail.utoronto.ca)）。如您在成都有其他需要，请联系蒋建军教授（电话：85501272 或邮箱：[jiangjianjun@scu.edu.cn](mailto:jiangjianjun@scu.edu.cn)）。您也可以联系指导本次研究的加拿大多伦多大学健康政策、管理与评估研究院的Peter C. Coyte教授（邮箱：[peter.coyte@utoronto.ca](mailto:peter.coyte@utoronto.ca)）

**同意参与研究及授权在保密的条件下使用访谈资料**

您以下的签名代表您授权我们对您进行访谈并在保密的条件下使用您的访谈资料作为这项研究的用途。您已得到此授权书的复印件。

| **参与者姓名（请清楚书写拼音）_______________________________________________** |
| --- |
| **参与者签名_________________________________________________________________** |
| **授权日期 __________________________________________________________________** |

**INFORMED CONSENT FOR PARTICIPANT INTERVIEW**

***The Lived Experience of Palliative Care Providers in Urban West China: A collaborative program with the West China School of Public Health and the Forth Teaching Hospital of Sichuan University, Chengdu, Sichuan Province, China.***

**Investigators:**

| **Rui Fu, MSc**  PhD student  Institute of Health Policy, Management and Evaluation  University of Toronto  1-647-879-6649 / 86-183-028-17609 (China)  [Rui.fu@mail.utoronto.ca](mailto:Rui.fu@mail.utoronto.ca) | **Lilian Jia Lu Lin, MPH**  PhD student  Institute of Health Policy, Management and Evaluation  University of Toronto  1-437-345-6807  [jialu.lin@mail.utoronto.ca](mailto:jialu.lin@mail.utoronto.ca) |
| --- | --- |
| **Jie Pan, PhD**  Professor and Vice Dean  West China School of Public Health  Sichuan University  86-28-85501272  [Panjie.jay@scu.edu.cn](mailto:Panjie.jay@scu.edu.cn) | **Jian Jun Jiang, MD, PhD**  Associate Chief Physician  Department of Palliative Medicine  The Forth Teaching Hospital of Sichuan University  86-28-85501520  [jiangjianjun@scu.edu.cn](mailto:jiangjianjun@scu.edu.cn) |
| **Peter C. Coyte, PhD**  Professor  Institute of Health Policy, Management and Evaluation  University of Toronto  1-416-978-8369  [Peter.coyte@utoronto.ca](mailto:Peter.coyte@utoronto.ca) |  |

**Purpose of the Project**

The purpose of this research is to ask about your experiences as a care provider at the Department of Palliative Medicine at the Forth Teaching Hospital of Sichuan University. The research aims to better understand the duties and responsibilities of a palliative care provider and how your interaction of patients and their family members have impacted your career perspective, health condition and quality of life. We are interested in learning about your thoughts on your job, why you chose to practice palliative care, and how you manage your work and personal life. We ask you to take part in this interview because you are currently working as a palliative care physician or a palliative care nurse at the Forth Teaching Hospital.

**Explanation of Interview:**

If you agree to participate in this research, the following will occur:

- Interviews will be conducted in person.
- The interview will be in a private room at the Forth Teaching Hospital of Sichuan University.
- We will record what you say and write it down. You will have a chance to listen to the tape of your interview and ask to remove anything you said. All interviews will be deleted when the research study is over.
- You will be asked to talk about what you think about the palliative care services you are providing and any experiences of interacting with palliative care recipients and their family caregivers.
- You will be asked to talk about the impact of being a palliative care provider on your physical and mental health status as well as your day-to-day life.
- The interview will last approximately 45-60 minutes.

**Confidentiality**

In order to protect you, any information collected in this study will stay private. All research materials will be kept in a locked office at the West China School of Public Health in Sichuan University. All electronic data will be kept in a password-protected document on a password-protected computer. All interviews will be conducted in a private room. Your answers will never be linked to your name and will never be used in a way that could identify you.

**Risks and Discomforts**

Your identity as a participant will not be told to people other than the investigators listed above. You do not have to answer any questions that make you uncomfortable. We do not anticipate that this research study has any risks other than discussions of sensitive topics related to your past interaction with patients and their family members. In the event that these discussions cause you discomfort or distress, we will provide a social work referral to address your concerns or needs.

**Benefits**

The results from this study will offer crucial insights on the operation of a comprehensive palliative care program in urban West China from a care provider’s perspective and help guide future development of similar care programs.

**Freedom to Withdraw Participation**

It is your choice to be in this research study. You can stop at any time and withdraw from this research study. If you withdraw from the study, we will delete all information related to your data. Your work at the Department of Palliative Medicine at the Forth Teaching Hospital of Sichuan University will not be affected if you decide not to be in the research study.

**Contact Information**

If you have concerns about this research or would like to have a copy of the results (in English or Chinese) after we have completed the project, please contact Rui Fu at [rui.fu@mail.utoronto.ca](mailto:rui.fu@mail.utoronto.ca). For local support in Chengdu, Sichuan Province, please contact Dr. Jian Jun Jiang at 86-28-85501272 or [jiangjianjun@scu.edu.cn](mailto:jiangjianjun@scu.edu.cn). You can also contact Professor Peter C. Coyte, supervisor of this research at the University of Toronto [peter.coyte@utoronto.ca](mailto:peter.coyte@utoronto.ca)

Your signature below shows that you understand the above and agree to participate in this interview. You have been given a copy of this consent form to keep.

| Please print your name ________________________________________ |  |
| --- | --- |
| Please sign your name ________________________________________ | Date ______________________ |
| Witness signature _____________________________________________ | Date ______________________ |

**Appendix S3** Interview guide (translated into English)

**Demographic information**

1. Are you a native of Chengdu, Sichuan province?

2. If you wouldn’t mind me asking, how old are you?

3. If you wouldn’t mind me asking, what is your current marital status? Do you have any children?

4. In your educational experience, have you ever received training or participated in courses that are related to palliative and hospice care?

a. What is your medical specialty?

5. Have you worked in other medical specialties before joining the Palliative Medicine Department (henceforth the Department)?

a. If yes, in what medical specialty have you worked in the past? Was it related to palliative and/or hospice care?

6. How long have you been working at the Department?

a. If you have worked in other departments of the West China Fourth Hospital before joining/being transferred into the Palliative Medicine Department, how long have you worked at the West China Fourth Hospital altogether?

**Work routine**

7. Can you tell me about how you first joined the Department?

a. If this is your first job, how did you find out about this Department at the West China Fourth Hospital? What made you become interested in palliative and hospice medicine? What concerns did you have when you first joined the Department?

b. If this is not your first job, how did you become aware of this Department? Why did palliative and hospice medicine interest you? How did you eventually decide to work here?

8. Can you briefly describe to me what a typical workday is like for you?

a. When do you usually get up in the morning?

b. What mode of transportation do you use to get to work (subway, bus, driving, walking)? Do you live in Chengdu? How long does it take for you to get to work?

c. What is the first thing that you do when you come to the Department each workday? What are the most important things that you do at work in the morning?

d. How do you usually arrange your lunch break? Do you have a fixed time to eat lunch and take a break?

e. What do you usually work on in the afternoon? What is it like when you are the busiest?

f. Where do you usually eat dinner? Is there a fixed time that you leave work? What is the last thing you need to do before leaving work?

9. How many patients are currently on your caseload?

**Patients**

10. Can you share with me about your most memorable patient(s)?

Probe: This may be a patient who is currently under your care, or a patient that you have cared for previously. You don’t need to tell me his/her name.

a. How old was the patient when s/he was admitted to the Department? What was the patient’s diagnosis/condition? What was the stage of the patient’s illness?

b. Did the patient’s child(ren) accompany him/her to the Department? If not, who did? Did the patient’s child(ren) visit him/her?

c. Could you communicate with the patient easily?

d. Could you communicate with the patient’s caregivers (children or other relatives or friends) easily?

e. What makes this patient so memorable to you?

**Perceptions of palliative and hospice care**

11. What do you think is the most unique aspect of palliative and hospice medicine compared to other departments in the hospital?

Probe: What do you think sets this Department apart from the rest? As a doctor/nurse at the Department, how is your job different from that of doctors/nurses in other departments?

a. Compared to intensive care? Compared to geriatrics? Compared to oncology?

b. What is the most distinctive aspect of your job compared to a doctor (or a nurse) in intensive care? Compared to geriatrics? Compared to oncology?

12. What do you think is the most fulfilling part of your work?

13. What is the most stressful part of your work?

a. How do you cope with work stress?

b. What do you think the hospital can do to help you relieve work stress?

c. What kind of policies do you think the government can implement to help you relieve work stress?

14. What do you think is the most important quality that a palliative and hospice care provider should possess?

a. What kind of person do you think would not be suitable for this job? Why?

b. What is the most difficult or challenging aspect of this job?

c. If there is a doctor (or nurse) who is about to step into the role of a palliative and hospice care provider, what do you think would surprise him/her the most about this job? What is one piece of advice that you would offer him/her?

15. In Canada, palliative care used to be called “end-of-life care” or “hospice care.” How important do you think is the term that we use?

a. Do you think the term “palliative care” is appropriate to be used in China?

b. Is there a more appropriate term that could be used to describe this medical specialty? Do you think that would be more acceptable to patients and families?

(Probes: conservative care? supportive care?)

**Personal life**

16. What are your family members’ attitudes toward your work?

a. Do you have children? How did you describe your work to them?

Probe: Did you only tell your children that you are a doctor/nurse or were you more specific and told them that you are a palliative care doctor/nurse?

b. Does your child’s teacher know about your job?

c. Have you ever faced an issue in your interactions with your family members because of your profession?

(Probe: When your family members found out about the nature of your job, did they raise any concerns or complaints? Can you give me an example?)

17. Have you ever discussed your work with your friends?

a. Does any of your friends know that you are engaged in palliative and hospice care? What was the situation that prompted you to tell him/her that?

b. What are your friends’ attitudes toward your work?

c. Have you ever faced an issue in your interactions with your friends because of your profession?

(Probe: When your friends found out about the nature of your job, did they raise any concerns or complaints? Did your job ever affect your social life outside of work? Can you give me an example?)

18. What do you think is the greatest learning you’ve gained from being a palliative and hospice care provider?

(Probes: importance of spending time with family, having a healthy lifestyle, meaning of life)

19. Someday in the future, if someone close to you, say a family member or a friend, had a terminal illness, would you suggest that they receive palliative and hospice care services?

20. As you may have heard, there is a severe shortage of medical professionals in the palliative and hospice care specialty.

a. What do you think might be the biggest reason for this?

Probe: Do you think that the public holds bias or prejudice against palliative and hospice care? Are there pressures coming from these medical professionals’ family and friends? Might it be related to the strained patient-provider relationship (i.e., lack of public trust in the hospital system and medical staff)?

b. What strategies do you think might promote medical students and other medical professionals to join the palliative and hospice care specialty?

21. Is there anything else you’d like to share with me?

**Ending**

Thank you very much for your time!
